# Gradient boosting reveals spatially diverse cholesterol gene signatures in colon cancer

**DOI:** 10.3389/fgene.2024.1410353

**Published:** 2024-11-29

**Authors:** Xiuxiu Yang, Debolina Chatterjee, Justin L. Couetil, Ziyu Liu, Valerie D. Ardon, Chao Chen, Jie Zhang, Kun Huang, Travis S. Johnson

**Affiliations:** ^1^ Department of Biostatistics and Health Data Science, Indiana University School of Medicine, Indianapolis, IN, United States; ^2^ Department of Medical and Molecular Genetics, Indiana University School of Medicine, Indianapolis, IN, United States; ^3^ Department of Statistics, Purdue University, West Lafayette, IN, United States; ^4^ Department of Biomedical Informatics, Stony Brook University, Stony Brook, NY, United States; ^5^ Indiana University Melvin and Bren Simon Comprehensive Cancer Center, Indianapolis, IN, United States; ^6^ Indiana Biosciences Research Institute, Indianapolis, IN, United States

**Keywords:** colon cancer (CC), cholesterol, bile acids, prognostic genes, machine learning (ML), spatial transcriptomics (ST)

## Abstract

Colon cancer (CC) is the second most common cause of cancer deaths and the fourth most prevalent cancer in the United States. Recently cholesterol metabolism has been identified as a potential therapeutic avenue due to its consistent association with tumor treatment effects and overall prognosis. We conducted differential gene analysis and KEGG pathway analysis on paired tumor and adjacent-normal samples from the TCGA Colon Adenocarcinoma project, identifying that bile secretion was the only significantly downregulated pathway. To evaluate the relationship between cholesterol metabolism and CC prognosis, we used the genes from this pathway in several statistical models like Cox proportional Hazard (CPH), Random Forest (RF), Lasso Regression (LR), and the eXtreme Gradient Boosting (XGBoost) to identify the genes which contributed highly to the predictive ability of all models, ADCY5, and SLC2A1. We demonstrate that using cholesterol metabolism genes with XGBoost models improves stratification of CC patients into low and high-risk groups compared with traditional CPH, RF and LR models. Spatial transcriptomics (ST) revealed that SLC2A1 (glucose transporter 1, GLUT1) colocalized with small blood vessels. ADCY5 localized to stromal regions in both the ST and protein immunohistochemistry. Interestingly, both these significant genes are expressed in tissues other than the tumor itself, highlighting the complex interplay between the tumor and microenvironment, and that druggable targets may be found in the ability to modify how “normal” tissue interacts with tumors.

## 1 Introduction

With an estimated 106,970 new cases and 52,550 deaths in the United States in 2022, colon cancer (CC) is the fourth most common cancer and second leading cause of cancer death (Siegel et al., 2023). CC staging is strongly tied to prognosis, where earlier stages have a much higher chance of long-term survival (Haggar and Boushey, 2009). However, symptoms such as bowel obstruction and the presence of bloody stools (hematochezia) are infrequently observed in the early stages of colorectal cancer (CC), resulting in many patients being overlooked until the disease has advanced to later stages (Zhang et al., 2021). Ultimately this makes it challenging to diagnose colon cancer at an early stage (Brenner et al., 2015; Wong et al., 2016). Despite recent advances in testing and treatment, the overall prognosis for patients with CC remains poor due to the lack of biomarkers for early detection and risk stratification of patients (Keum and Giovannucci, 2019). High molecular heterogeneity is a hallmark of CC, and studies have shown that this heterogeneity is associated with differences in survival and response to therapy among patients with the disease (Burrell and Swanton, 2014; Dienstmann et al., 2017).

Thus, to address the heterogeneity in patients that drive their differences in survival and response to therapy, it is important to explore valuable and unifying diagnostic and prognostic factors to guide the development therapeutics that would be effective for this broad and varied patient population (Schork, 2015). Recent evidence has shown that a high-cholesterol diets are strongly associated with an increased risk for CC (Wu et al., 2022), and it has been shown that a diet-responsive phospholipid-cholesterol axis regulates intestinal stem cell (ISC) proliferation and tumorigenesis (Wang et al., 2018). CC with high levels of cholesterol synthesis may have a high chance of cancer recurrence and worse progression or relapse-free survival (Xie et al., 2022). Recent studies have revealed that cholesterol plays a more prominent role in the advanced stages of colorectal cancer, rather than during the early stages of the disease (Wu et al., 2022). The goal of our research was to determine whether these clinico-molecular associations can be leveraged to predict prognosis, determine where in the tumor and microenvironment these genes are expressed, and the implications for developing therapeutic targets.

Multi-omics sequencing data has begun to change the traditional methods used to stratify cancer patients and identified promising therapeutic avenues (Cancer Genome Atlas Network, 2012; Zhao et al., 2014). However, the inherent characteristics of omics data, such as high dimensionality, small sample size, and category imbalance, usually pose significant computational challenges (Boulesteix and Strimmer, 2007). Fortunately, the rapid development of machine learning (ML) algorithms has occurred in parallel, and these algorithms have been widely applied in the diagnostic classification and prognosis of disease (Camacho et al., 2018). ML complements traditional statistical methods for improving cancer diagnosis, detection, prediction, and prognosis by including more complex interactions and frequently improving performance at the cost of interpretability and potentially, external validity (Hijazi and Chan, 2013). Many ML approaches are applied to deal with biological multi-omics data of high-dimensional samples (Arjmand et al., 2022). One such algorithm called gradient boosting decision trees (i.e., XGBoost), is often more accurate in cancer research than other machine learning algorithms, like RF, SVM, and logistic regression (Islam et al., 2020).

The XGBoost model has been shown to be highly effective in predicting cancer outcomes, outperforming other machine learning algorithms and achieving high accuracy and specificity. This model allows researchers to identify complex relationships between phenotypes, gene expression, and predict patient outcomes more accurately. This study aimed to investigate genes associated with cholesterol metabolism and their association with CC risk and clinical outcomes. First, we trained a novel XGBoost model that can be used for patient risk stratification and performs well compared to other established methods. Then, we used spatial transcriptomic and proteomic data to visualize the gene expression of prognostic genes from the XGBoost model to study the distribution of these genes in tumor tissue, identifying the cellular and spatial context of these genes within the tumor microenvironment, such as the expression levels in tumor cells, stromal cells, as well as their location within different regions of the tumor tissue.

## 2 Materials and methods

### 2.1 Data preparation

**FIGURE 1:**
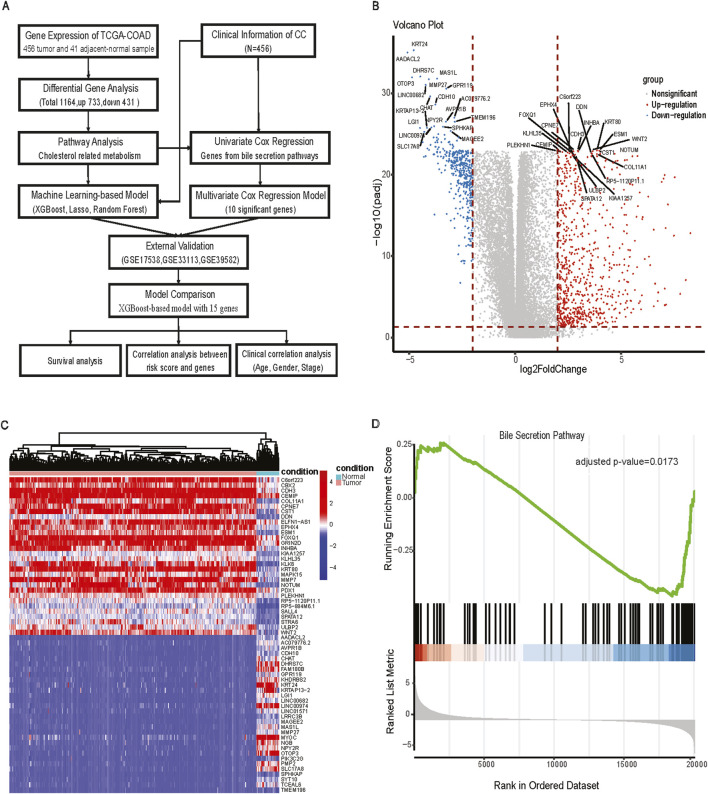
The workflow for analysis and differentially expressed analysis between tumor and adjacent normal tissue. **(A)** The workflow of processing and analyzing the data; **(B)** Volcano plot of Differentially expressed genes (FDR <0.05, |log2FC|>2); **(C)** Heatmap of differentially expressed genes between tumor and adjacent normal tissues; **(D)** GSEA plot of bile secretion pathway.

RNA-seq raw counts were retrieved from The Cancer Genome Atlas (TCGA) (https://portal.gdc.cancer.gov/projects) to study the relationship between cholesterol and CC prognosis. TCGA-COAD (N = 512) (Cancer Genome Atlas Network, 2012) read counts were normalized with the transcripts per million (TPM) method. After the data filtering process by removing the duplicates for each patient, 456 CC samples and 41 adjacent-normal tissues with survival information, age, gender, and stage were included for further analysis. The three Gene Expression Omnibus microarray datasets (https://www.ncbi.nlm.nih.gov/geo/) were used for external validation cohorts: GSE17538 (N = 232) (Smith et al., 2010), GSE33113 (N = 90) (Felipe de Sousa et al., 2011), and GSE39582 (N = 566) (Marisa et al., 2013). The workflow for processing and analysing the data is shown in Figure 1A.

### 2.2 Differential gene expression analysis

DEG analysis between primary CC samples and normal tissues was performed using the Wilcoxon rank-sum test (Li et al., 2022). We used TCGA identifiers in the sample name to delineate tumor or adjacent-normal samples (primary tumor: 01A, adjacent normal: 11A). DEG significance was defined by an FDR adjusted *p*-value <0.05, and |log2FC| > 2. The upregulated and downregulated genes were visualized in volcano plots with EnhancedVolcano (Blighe et al., 2019). Heatmaps were generated with the pheatmaps package to show the expression profiles of DEGs (Kolde, 2012). DEGs were mapped to terms in the Kyoto Encyclopedia of Genes and Genomes database (KEGG) database and the Gene Ontology (GO) for functional enrichment and pathway analysis. The KEGG pathway enrichment analysis was performed with clusterProfiler (Yu et al., 2012). Enrichment results with a false discovery rate (FDR) < 0.05 were classified as significant functional categories.

### 2.3 CPH model construction and survival analysis

Univariate Cox regression was applied to examine the prognostic value of cholesterol-related DEGs in CC patients using the package survival (Therneau and Lumley, 2015). *P*-values <0.05 were considered significant. Genes with hazard ratios (HR) > 1 were called high-risk genes, while HR < 1 were called low-risk genes. Next, using the same survival R package, we constructed a multivariate Cox proportional hazard (CPH) model for cholesterol metabolism using the prognostic cholesterol-related genes of CC with log-rank *p*-value <0.05 from the univariate cox, to better understand the interactions between the significant univariate genes. The risk score for patient j was calculated from our Cox model described below:

$${risk\_score}_{j}=sigmoid\left(\sum_{i=1}^{n}\beta_{i}\times X_{i,j}\right)$$

Where, $\beta_{i}$ represents the coefficient for each gene in the multivariate cox regression model, and $X_{i,j}$ represents the expression of gene i in patient j . The survival data were obtained from the clinical metadata files. The median risk score was the cutoff to classify the CC patients into low-risk and high-risk groups. The evaluation indicator of the survival analysis was disease-free survival (DFS), also known as relapse-free survival (RFS), which refers to the length of time after the end of primary cancer treatment that the patient survives without any signs or symptoms of the tumor recurrence.

The model was used to evaluate the association between RFS and cholesterol-related genes. The model provided risk scores (hazard ratios) and subsequently patients were divided into a low-risk group and high-risk group, stratified by the median risk score. Kaplan-Meier log-rank analyses were performed using the survival package to understand the significance of relapse-free survival differences between these two groups generated by the CPH model.

### 2.4 Machine learning model

Several ML models were applied to our study of cholesterol pathway-based CC prognosis for comparison purposes: LR, RF, and XGBoost. These were implemented using the following packages: glmnet (Friedman et al., 2010), randomForest (Liaw and Wiener, 2002), and xgboost (Chen and Guestrin, 2016). Five-fold cross-validation was performed for each model to select the hyperparameters for the optimal ML model.

For each algorithm, ML-based models representing all combinations of identified biomarkers were built. We curated 75 cholesterol biosynthesis (KEGG bile secretion pathway, hsa04976) gene with available transcriptomics data in both TCGA and GEO datasets. Further SLC22A8 was excluded from downstream analysis since it was not expressed in more than 85% of samples in the TCGA-COAD dataset. Next, we used feature importance ranking to pick the top 20% features, and fifteen genes were selected based on the importance of the features in the ML models. This novel fifteen-gene signature was selected by feature importance ranking, and a series of external validations were performed using the previously mentioned microarray data. For XGBoost model, the primary hyperparameters for XGBoost include the number of trees (nrounds), maximum depth of each tree, subsample ratio, and gamma value. These hyperparameters play a pivotal role in determining the model’s behavior and performance. We conducted a five-fold cross-validation using the default hyperparameters of the XGBoost model on our training dataset. These default parameters are maximum depth = 6, subsample = 0.5, gamma = 0, and nrounds = 60. Then,we performed spatial visualization of the fifteen genes from XGBoost modelling using Seurat (Satija et al., 2015) and the 10X Genomics Visium platform to identify specific regions within the tumor that are associated with different biological processes or clinical outcomes. The spatial transcriptomics data is colon adenocarcinoma available from 10X Genomics (Ståhl et al., 2016) (https://www.10xgenomics.com/resources/datasets/human-intestine-cancer-1-standard). Histologic correlates in the transcriptomic data were identified. Immunohistochemistry data from the Human Protein Atlas (Uhlén et al., 2015) (https://www.proteinatlas.org/) was used to understand whether protein expression distribution matched aligned with findings from the spatial transcriptomics.

### 2.5 Model comparison

We compared our model with a published model, which uses eight immune-related genes to predict relapse-free survival in CC (Wen et al., 2020). We compared both models in the TCGA-COAD (training), GSE17538 (testing), GSE39582 (testing), and GSE33113 (testing) datasets, evaluated using the log-rank *p*-value based on the risk scores from each model. In our study, we evaluated and then compared the performance of the traditional CPH model with ML models in predicting RFS or DFS in CC. Our results showed that a more complex gradient boosting ensemble model, like XGBoost, can improve patient stratification and highlights the prognostic potential of cholesterol pathways in CC.

Furthermore, since our results showed superior performance of XGBoost model and compared to the other models for the various validation datasets, we included age and cancer stage as covariates. Age at diagnosis was directly extracted from the dataset and incorporated as a continuous variable. Cancer stages were simplified into four broad categories: Stage I (including IA, IB), Stage II (including IIA, IIB, IIC), Stage III (including IIIA, IIIB, IIIC), and Stage IV (including IVA, IVB). This allowed for clearer stage groupings in the analysis, facilitating more robust comparisons across the validation datasets.

### 2.6 Statistical analyses

All the statistical analyses were conducted with R software (version 4.2.1). Significance was determined at the following levels *p* < 0.05 (*), *p* < 0.01 (**), and *p* < 0.001 (***). Unless otherwise noted, statistical testing was conducted using base R implementations (Ihaka and Gentleman, 1996).

## 3 Results

### 3.1 Differential gene expression analysis reveals enrichment of the bile section pathway

TCGA-COAD identified a total of 1,164 DEGs, of which 733 were upregulated and 431 were downregulated in CC tissues compared with adjacent normal tissues. There were numerous DEGs with high fold changes and low *p*-values (Figure 1B), the expression changes of DEGs clearly distinguished CC tissues and adjacent-normal tissues (Figure 1C). Among GO and KEGG pathway analyses for downregulated genes, only Bile Secretion was enriched (Figure 1D, GSEA adjusted nominal *p*-value = 0.0173; hypergeometric test adjusted *p*-value = 0.0063). Bile secretion plays a key role in cholesterol homeostasis. We extracted 75 genes from the Bile secretion pathway using KEGG/GO methods for downstream prognostic model construction.

### 3.2 Construction of cholesterol related prognostic model for colon cancer

**FIGURE 2:**
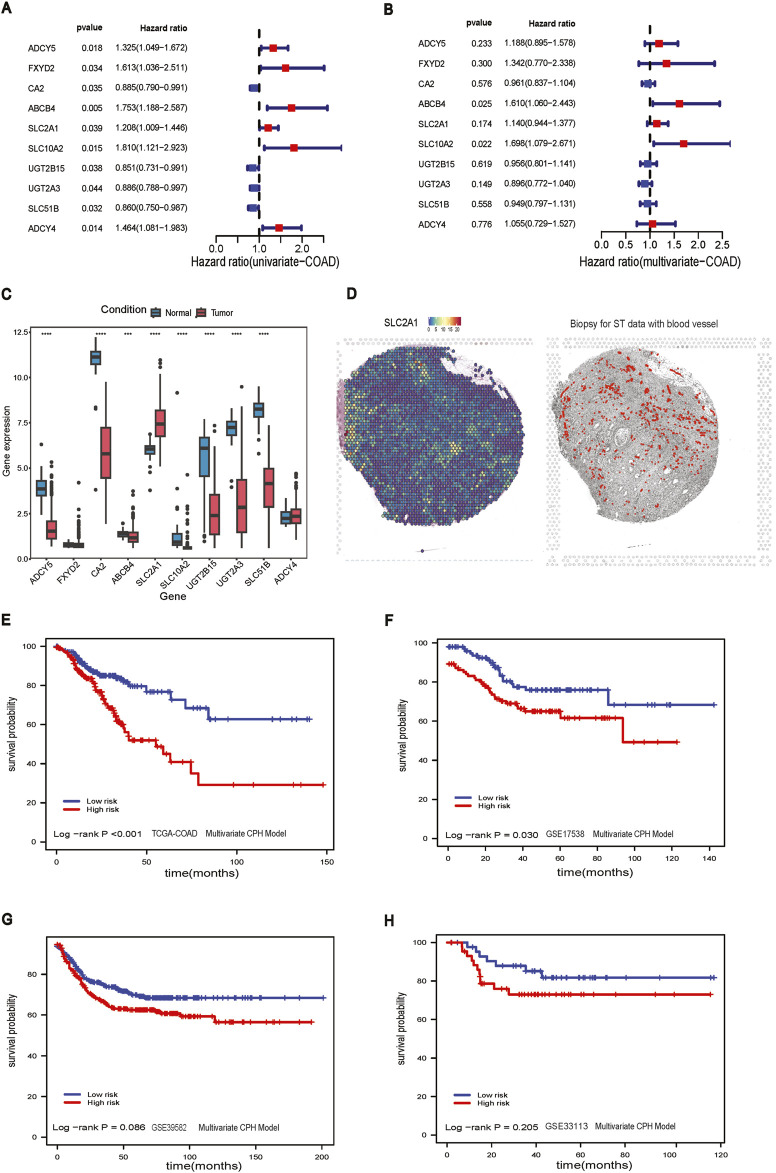
CPH model construction. **(A)** Forest plot of univariate CPH model with significant genes; **(B)** Forest plot of multivariate CPH model; **(C)** boxplot of genes in multivariate CPH model; **(D)** Left panel: Spatial visualization of SLC2A1 gene expression; Right panel: The histological biopsy with blood vessels for ST data; **(E)** Survival analysis of multivariate CPH model in TCGA-COAD dataset; **(F)** Survival analysis of multivariate CPH model in GSE17538 dataset; **(G)** Survival analysis of multivariate CPH model in GSE39582 dataset; **(H)** Survival analysis of multivariate CPH model in GSE33113 dataset.

From the univariate CPH analysis, ten genes were found to be statistically significant, including ADCY5, FXYD2, CA2, ABCB4, SLC2A1, SLC10A2, UGT2B15, UGT2A3, SLC51B, and ADCY4 (Figure 2A). Meanwhile, we performed the Benjamini-Hochberg FDR correction and Bonferroni method to get the corresponding adjusted *p*-value for multiple comparison testing (Supplementary Table S1). Then, we constructed a multivariate prognostic CPH model using these ten genes (Figure 2B). Eight out of these ten genes were statistically significant between tumor and adjacent normal samples (Figure 2C). Of the genes that were significant, only SLC2A1 had a higher gene expression in the tumor samples than in adjacent-normal samples. The genes that were significant in the multivariate CPH model but were downregulated in the tumor compared to adjacent-normal tissues in the bulk RNA-seq were: ADCY5, FXYD2, CA2, ABCB4, SLC10A2, UGT2615, UGT2A3, SLC51B, ADCY4. The spatial visualization of SLC2A1 gene expression in intestine cancer spatial transcriptomics data showed a non-uniform distribution in the tissue and SLC2A1 (GLUT1) expression tended to colocalize with regions that had small blood vessels penetrating the tumor (Figure 2D). The final CPH model consisted of:

$$\begin{array}{c} {risk\_score}_{CPH}=sigmoid\;(0.173*ADCY5+0.294*FXYD2\\ -0.039*CA2+0.476*ABCB4+0.131*SLC2A1\\ +0.529*SLC10A2-0.045*UGT2B15\\ -0.11*UGT2A3-0.052*SLC51B+0.054*ADCY4) \end{array}$$

The total of 456 CC patients were divided into a high-risk group (n = 228) and a low-risk group (n = 228) based on the median risk score of 0.842. The Kaplan-Meier analysis in the TCGA-COAD dataset confirmed that the RFS stratification performance of CC patients had shown statistically significant in the high-risk group and the low-risk group (log-rank *p*-value <0.001), and the high-risk group had a worse overall RFS compared to those in the low-risk group (Figure 2E). To further explore the performance of the multivariate CPH model using external GEO datasets, we applied it to GSE17538 (log-rank *p*-value = 0.030), and RFS performance of CC patients showed a significant difference between the low and high-risk group (Figure 2F). The CPH model was also evaluated in GSE39582 (log-rank *p*-value = 0.086) and GSE33113 (log-rank *p*-value = 0.205), but neither dataset showed a statistical difference between the low and high-risk groups (Figures 2G, H).

### 3.3 Machine learning identifies prognostic genes with varying predictive power across colon cancer datasets

We used 75 genes from the bile secretion pathway related to cholesterol and several cross-validated ML methods to identify genes that were consistently prognostic (Supplementary Table S2). We used Random Forests, Lasso regression, and XGBoost feature importance outputs to identify the most important genes for predicting prognosis. To improve the accuracy and interpretability of ML model by focusing on the most important features, we identified fifteen genes as being the most important for stratifying patients. We further identified two genes that were common to all models as being high importance and prognostic (SLC2A1 & ADCY5). Rather than fixing the genes used in each predictive model, the purpose of using multiple machine learning models is to regularize the associations between genes and prognosis, identifying only highly consistent genes.

**FIGURE 3:**
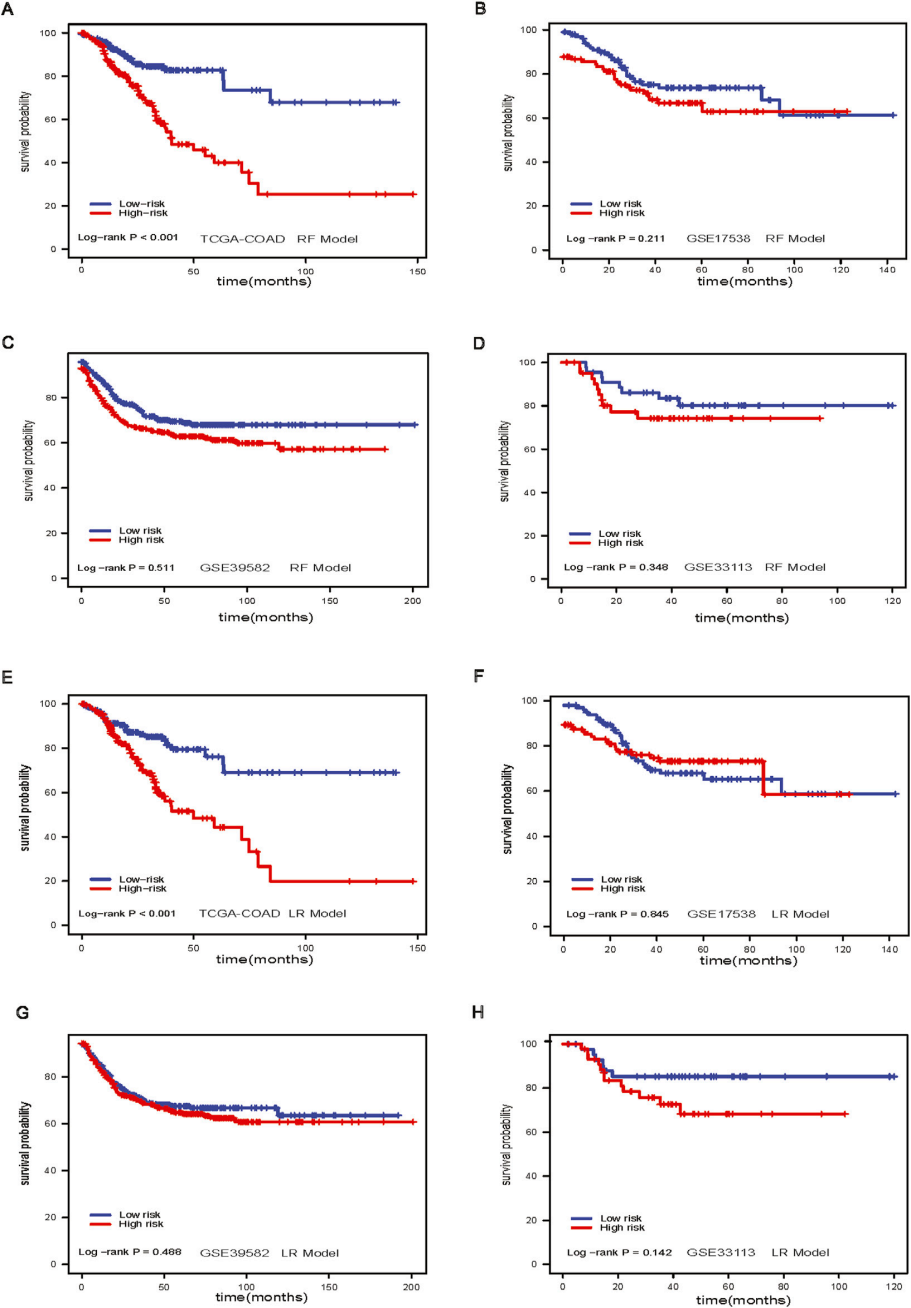
Random Forest model and Lasso Regression model. **(A)** Survival analysis of RF model in TCGA-COAD dataset; **(B)** Survival analysis of RF model in GSE17538 dataset; **(C)** Survival analysis of RF model in GSE39582 dataset; **(D)** Survival analysis of RF model in GSE33113 dataset; **(E)** Survival analysis of Lasso model in TACG-COAD dataset; **(F)** Survival analysis of lasso model in GSE17538 dataset; **(G)** Survival analysis of lasso model in GSE39582 dataset; **(H)** Survival analysis of Lasso model in GSE33113 dataset.

The RF model stratified patients with significant differences in survival in the TCGA-COAD dataset (log-rank *p*-value <0.001, Figure 3A). However, the results of RF models did not differentiate low-risk and high-risk groups in GSE17538 (log-rank *p*-value = 0.211, Figure 3B), GSE39582 (log-rank *p*-value = 0.511, Figure 3C), and GSE33113 (log-rank *p*-value = 0.348, Figure 3D). The LR model, like the RF model, stratified patients in the TCGA-COAD (log-rank *p*-value <0.001, Figure 3E). However, the model performance was not significant in GSE17538, GSE39582 and GSE33113 (Figures 3F–H).

**FIGURE 4:**
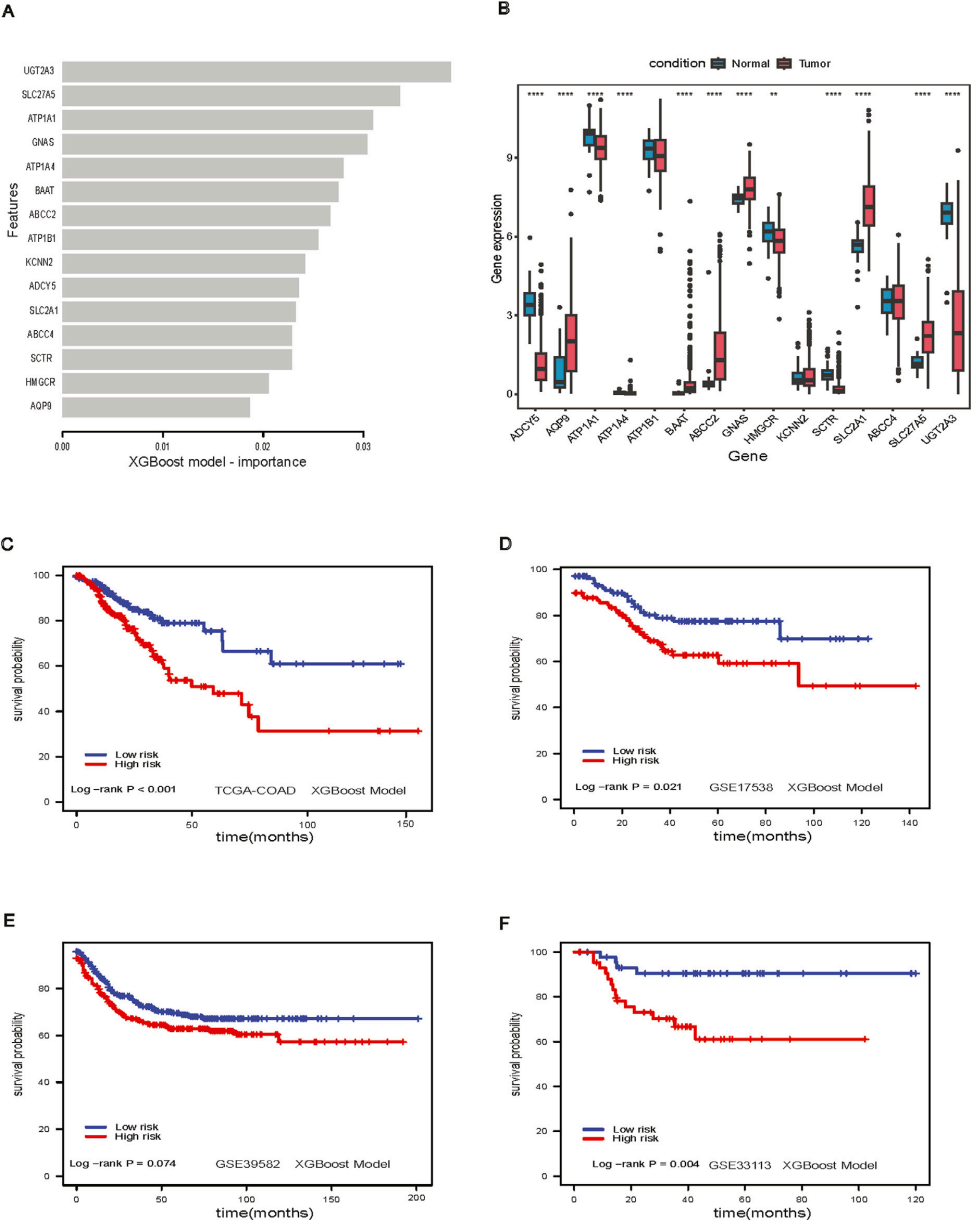
XGBoost Model. **(A)** Feature importance in XGBoost model; **(B)** Boxplot of important genes expression in XGBoost model; **(C)** Survival analysis of XGBoost model in TCGA-COAD dataset; **(D)** Survival analysis of XGBoost model GSE17538 dataset; **(E)** Survival analysis of XGBoost model in GSE39582 dataset; **(F)** Survival analysis of XGBoost model in GSE33113 dataset.

We examined the feature importance for the XGBoost model (Figure 4A). Twelve out of fifteen genes were found to be significantly differentially expressed between adjacent-normal and tumor tissues in the TCGA-COAD dataset (Figure 4B). The XGBoost model stratified low-risk and high-risk groups in the TCGA-COAD dataset (*p*-value <0.001, Figure 4C). Patients could be stratified significantly in GSE17538 (log-rank *p*-value = 0.021, Figure 4D), nearly significantly in GSE39582 (log-rank *p*-value = 0.07, Figure 4E) and significantly in GSE33113 (log-rank *p*-value = 0.004, Figure 4F). For the XGBoost model, we further analyzed the patient characteristics, including stage, sex, and age (Supplementary Table S3), and the showing differences in stage across risk groups (*p*-value <0.001, Supplementary Figures S1A, B). Notably, SLC2A1 gene expression was higher in tumors (Figure 4B), we further investigated this gene in the spatial transcriptomics.

For the spatial visualization of RNA and gene expression, we found that the prognostic genes from XGBoost model, especially GNAS (Supplementary Figure S2A), ATP1A1 (Supplementary Figure S2B), ATP1B1 (Supplementary Figure S2C), colocalized with the densest regions of tumor. The Protein atlas IHC staining also colocalized to tumor regions. SLC2A1 had a distinct distribution, being less enriched in tumor regions, and instead favoring regions with blood vessels. This aligns with SLC2A1’s known role in transporting glucose across endothelial cells and in red blood vessels (Zheng et al., 2010; Paikari et al., 2019) (Supplementary Figure S2D). ADYC5 had a stromal pattern in both the transcriptomics and IHC (Supplementary Figure S3A). HMGCR, the rate-limiting enzyme in cholesterol synthesis, showed a mixed tumor/stromal pattern in both the transcriptomic and proteomic data (Supplementary Figure S3B). The spatial distribution of KCNN2 and SCTR (Supplementary Figures S3C, D) were difficult to discern in the transcriptomic data due to low expression. The intensity of the proteomic data was also much more muted compared to the other genes in this analysis.

### 3.4 XGBoost outperforms other models in prognostic accuracy across multiple colon cancer datasets

**FIGURE 5:**
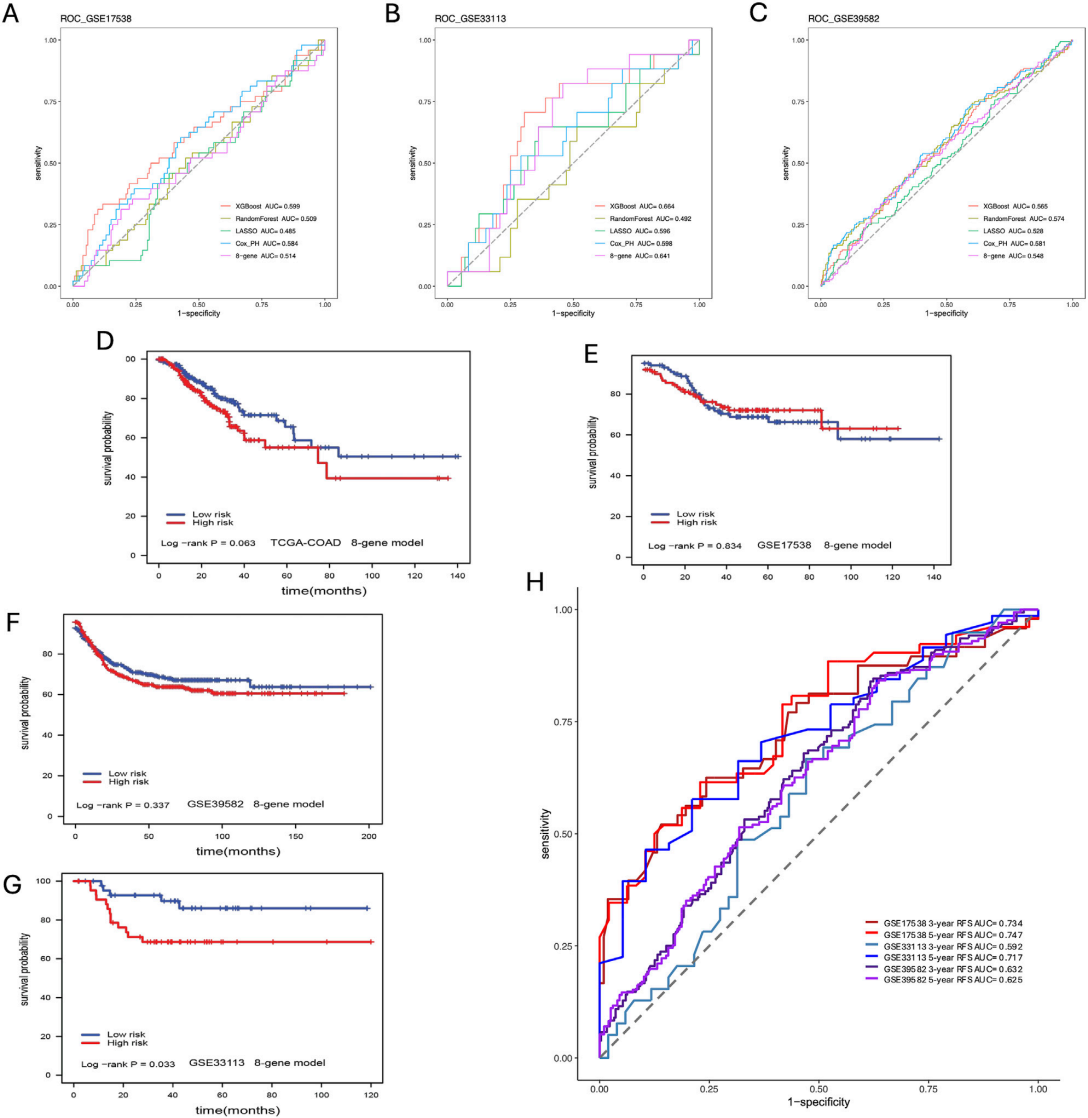
Model Comparison. **(A)** ROC curve analysis of models for prediction of CC patients with 3-year survival in GSE17583; **(B)** ROC curve analysis of models for prediction of CC patients with 3-year survival in GSE33113; **(C)** ROC curve analysis of models for prediction of CC patients with 3-year survival in GSE39582; **(D)** Eight-gene model validation in TCGA-COAD dataset; **(E)** Eight-gene model validation in GSE17538 dataset; **(F)** Eight-gene validation in GSE39582 dataset; **(G)** Eight-gene validation in GSE33113 dataset; **(H)** ROC curve analysis of models including additional covariates for prediction of CC patients with 3- and 5-year survival in all validation datasets.

We compared our CPH, RF, LR, and XGBoost models’ performance against a previously published eight-gene panel. We performed the survival analysis for each model to get the log-rank *p*-value. From the results of the model comparison (Table 1), we can conclude that XGBoost performed better than LR, RF, CPH and eight-gene models in the TCGA-COAD dataset (*p*-value <0.001) in GSE17538 (*p*-value = 0.021), and in GSE33113 (*p*-value = 0.004). To further compare the performance of ML models we built, we also used receiver operating characteristic (ROC) curve to evaluate the high and low risk classification tasks with CPH, RF, LR, XGBoost and eight-gene models with 3-year survival. For validation dataset GSE17538, the XGBoost model had the receiver operating characteristic area under the curve (AUC) 0.599, which was better than the other models (Figure 5A); and for validation dataset GSE33113, the XGBoost model had AUC 0.664, which performed better (Figure 5B). None of the models—XGBoost, LR, RF, or CPH—achieved an AUC above 0.590 on the GSE39582 dataset. The AUC for the XGBoost model on the GSE39582 dataset was 0.565 (Figure 5C), which was comparable to the poor performance of the other models. However, given that all models showed similarly low performance, we are cautious about drawing conclusions from this dataset using gene expression alone. We also used the eight genes and corresponding coefficients from the published paper to build the comparison model. This comparator model could not significantly stratify the TCGA-COAD, GSE17538, or GSE39582 patients (Figures 5D–F) but could significantly stratified patients in GSE33113 (Figure 5G, log-rank *p*-value = 0.033). Other metrics for evaluation of model performance such as sensitivity, specificity, F1-score, precision-recall AUC (PR-AUC) and AUC are provided in Supplementary Table S4.

**TABLE 1:** Model comparison.

Dataset	Index	Classification	XGBoost	LR	RF	CPH	8-gene panel
TCGA-COAD (N = 456)	p-value	Training	<0.001^***^	<0.001^***^	<0.001^***^	<0.001^***^	0.063
GSE 17538 (N = 232)	p-value	Validation	0.021^*^	0.845	0.210	0.030^*^	0.834
GSE 33113 (N = 90)	p-value	Validation	0.004^**^	0.142	0.348	0.205	0.033^*^
GSE 39582 (N = 566)	p-value	Validation	0.074	0.488	0.051	0.086	0.337

Note: XGBoost, eXtreme Gradient Boosting; LR, Lasso Regression; RF, Random Forest; CPH, Cox Proportional Hazard; TCGA, The Cancer Genome Atlas.

The symbols *, **, and *** indicate the level of statistical significance of the results, with * representing *p*-value <0.05, ** representing *p*-value <0.01, and *** representing *p*-value <0.001.

Due to the superior performance of the XGBoost model in these baseline comparisons across various validation datasets, we extended the model to include additional covariates such as age, and disease stage. This enhancement resulted in not only higher PR-AUC and AUC values but also improvements in F1 scores, sensitivity, and specificity (Figure 5H; Supplementary Tables S4, S5). For instance, after incorporating these covariates, the XGBoost model achieved AUCs of 0.734 for GSE17538, 0.592 for GSE33113, and 0.632 for GSE39582 in predicting 3-year survival (Supplementary Table S5). Our method performed comparably to stage depending on the metrics and universally performed better in PR-AUC, which better accounts for imbalanced data. Note the lower performance in GSE33113 maybe due to all patients being Stage II. Despite the lower 3-year survival AUC in GSE33113, it highlights a distinct advantage that our method has over stage, i.e., in cases where cohorts lack stage heterogeneity, our method can still achieve a high 5-year AUC of 0.717 without diversity in the stage information. These findings further demonstrate that incorporating patient-specific information will overall boost model performance.

### 3.5 XGBoost-identified prognostic features in cholesterol metabolism reveal stage-specific survival predictions in colon cancer

**FIGURE 6:**
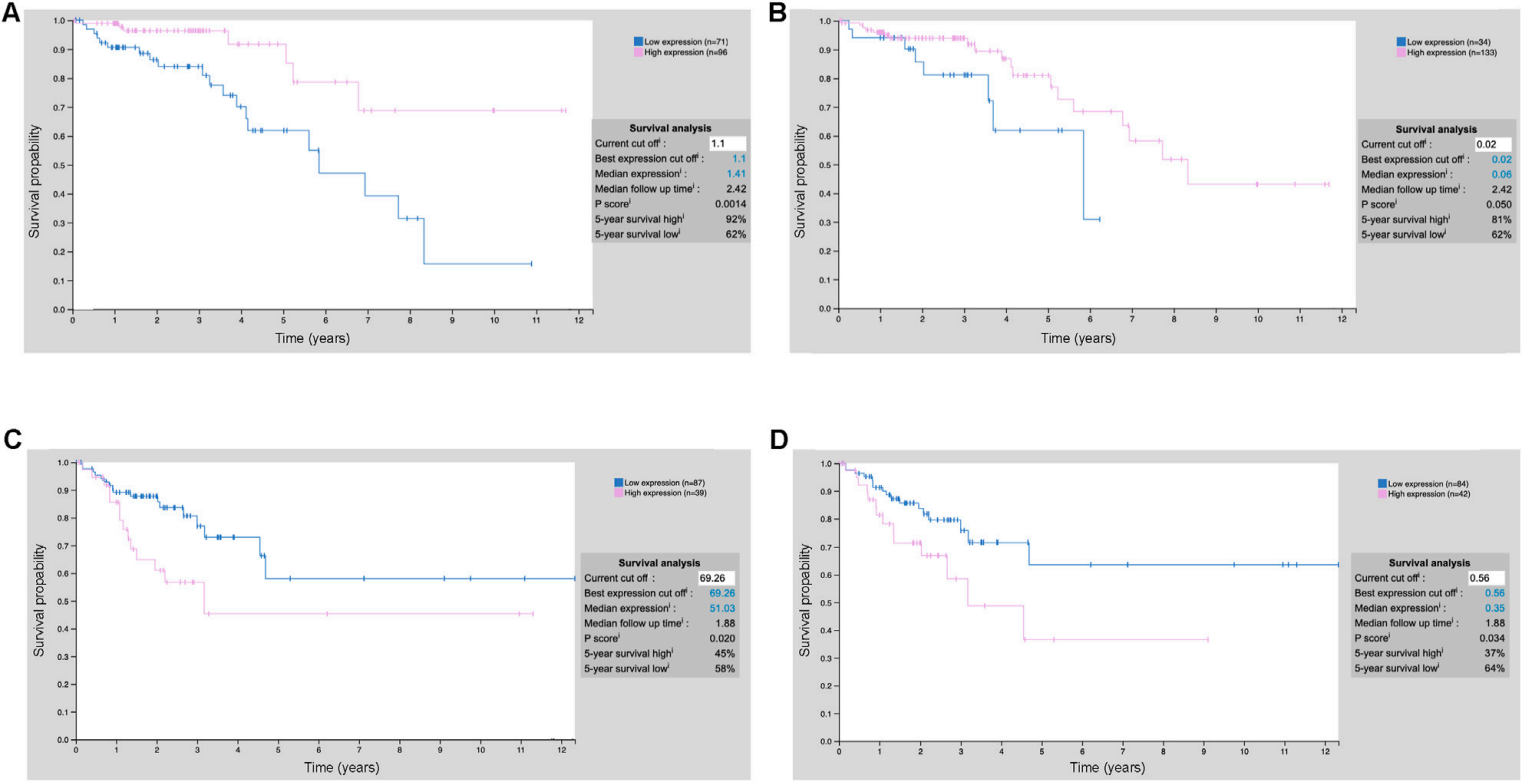
Prognostic values of the top model features using IHC staining. **(A)** UGT2A3 protein expression stratification COAD stage 2; **(B)** BAAT protein expression stratification COAD stage 2; **(C)** SLC2A1 protein expression stratification COAD stage 3; **(D)** ADCY5 protein expression stratification COAD stage 3.

The XGBoost model is used to identify key prognostic features related to cholesterol metabolism for further mechanistic study (Figure 4A). To demonstrate the prognostic potential and need for further study, we have included an additional external validation cohort from the Human Protein Atlas showing that numerous of our top prognostic features are predictive of survival during early and mid-stage COAD using IHC staining (Figure 6). Notably, during stage 2 COAD bile secretion related genes such as UGTA3 (Figure 6A) and BAAT (Figure 6B) are predictive of longer survival. In contrast, during stage 3 COAD cholesterol pathway genes such as SLC2A1 (Figure 6C) and ADCY5 (Figure 6D) are predictive of shorter survival. This highlights that even at the protein level these genes can be prognostic even at the early or mid-stages of COAD.

## 4 Discussion

In this study, we obtained the gene expression profiles from the TCGA-COAD and GEO datasets using multiple bioinformatics approaches. We then performed differential gene analysis and KEGG pathway analysis where only the bile secretion pathway was enriched in the KEGG downregulation pathway analysis. Bile secretion is an integral component of normal cholesterol metabolism. Several studies have suggested that bile acids have an important impact on the development and progression of colon cancer. One proposed mechanism is that bile acids promote inflammation and oxidative stress in colon cells, leading to DNA damage and mutations that may contribute to tumorigenesis (Degirolamo et al., 2011; Nguyen et al., 2018). Other studies have suggested that bile acids may promote cell proliferation and survival in colon cancer cells, further contributing to tumor growth, by altering the composition and function of cell membranes (Liu et al., 2016; Ocvirk and O’keefe, 2017; Hegyi et al., 2018).

We trained traditional CPH and machine learning models on the TCGA-COAD project and trained the models on three GEO datasets. Subsequently we extracted feature importance measures, investigated the expression of consistently prognostic genes in spatial transcriptomics and protein IHC. The XGBoost model performed better than the traditional CPH, LR, and RF models. We compared our 15-gene panel with a published 8-gene signature, and our XGBoost model performed better than other models. When comparing our XGBoost model to recently published studies, Du et al. (2022) reported an AUC of 0.606 for predicting 3-year survival using the GSE39582 dataset with Lasso as their final model. In contrast, after incorporating age and disease stage information, our XGBoost model achieved an AUC of 0.632 (Supplementary Table S5). Similarly, Liu X.-S. et al. (2022) reported an AUC of 0.552 for 5-year survival prediction using Lasso on the same dataset, with their microenvironment score (MES) high/low risk grouping yielding an AUC of 0.618. In comparison, our XGBoost model achieved an AUC of 0.625 for 5-year survival. Li et al. (2024) validated their Lasso model on an external dataset, reporting an AUC of 0.66 for 3-year survival. Our XGBoost model, however, produced AUCs as high as 0.734 on external validation datasets such as GSE17538. For 5-year survival, our model achieves even higher AUCs, up to 0.747. The PR-AUCs range from 0.49 to 0.63 for 3-year survival and increase further for longer survival periods. These results indicate that our XGBoost model demonstrates comparable or superior performance across multiple datasets, highlighting the potential advantages of using more flexible machine learning approaches like XGBoost in survival prediction tasks. It is worth noting even in the GSE33113 dataset of Stage II only patients we were still able to achieve AUC as high as 0.717 for 5-year survival signifying that the gene signature itself adds predictive value to the clinical features like stage. Next, we investigated which genes involved in cholesterol metabolism were most tied to CC prognosis.

Among our 15 genes panel, two (ADCY5, SLC2A1) were directly involved in cholesterol metabolism. Some cohort studies have associated the low expression of ADCY5 with a better prognosis in CC (Zhang et al., 2021). In our CPH model, ADCY5 was a high-risk feature, which aligns with these results. Moreover, several studies showed that SLC2A1 expression was higher in CC tissues and associated with worse overall survival. Our histological assessment demonstrated that SLC2A1 showed a distribution more restricted to vasculature. Thus, SLC2A1 may be a diagnostic and prognostic biomarker in CC (Liu Y. et al., 2022) related to tumor blood supply. Even at the protein level bile secretion and cholesterol pathway genes can be prognostic at the early or mid-stages of COAD. The potential role of ADCY5 in colorectal cancer prognosis is underscored by its methylation status and expression patterns observed in both type 2 diabetes mellitus (T2DM) (Wei et al., 2022) and glioblastoma studies (Can et al., 2024). In T2DM patients, elevated methylation levels of ADCY5 are associated with an increased risk of developing colon cancer, and in glioblastoma, ADCY5 functions as a tumor suppressor, implies that similar mechanisms could be at play in colorectal cancer. Thus, our model identifies important markers such as SLC2A1 and ADCY5 which can provide valuable prognostic information for colon cancer patients, potentially guiding treatment decisions.

However, in this study, we recognize several limitations of our model and propose directions for future research. The performance metrics, particularly the area under the curve (AUC), were not as high as we had hoped. One significant challenge we encountered was the missing patient information, which impeded our ability to create clinically usable models. While our primary objective was to identify genes and proteins that could serve as potential biomarkers or therapeutic targets, enhancing clinical utility necessitates addressing these data gaps. Future studies should consider incorporating additional patient information, such as epigenetic, electronic medical records (EMR), and genetic data, to improve the accuracy and predictive power of our model. Despite these limitations, our model successfully identified prognostic markers for colon cancer, particularly genes such as SLC2A1 and ADCY5, which are also supported by existing literature. This underscores the relevance of our findings in the broader context of colon cancer prognosis and highlights the potential for further exploration of these biomarkers in clinical applications. Addressing the identified gaps in future research will be crucial for enhancing the clinical applicability of our results and improving patient outcomes.

## 5 Conclusion

Our results showed that a more complex ensemble model, XGBoost, can improve patient risk stratification and highlight the prognostic potential of cholesterol pathways in CC. In fact, incorporating more patient-specific information such as age, stages of disease, etc. significantly boost model performance. Furthermore, we demonstrated that cholesterol-related genes might play a notable role in CC progression. ADCY5 expression was mostly found in stromal regions, and SLC2A1 coincided with blood vessels. Collectively, our results were consistent across several datasets, suggesting that ADCY5 and SLC2A1 could potentially serve as robust prognostic biomarkers for CC, and underscore a significant role played by the microenvironment in the progression of colon cancer.

## Data Availability

The original contributions presented in the study are included in the article/supplementary material, further inquiries can be directed to the corresponding author.
