# Supplementary material for: Gradient boosting reveals spatially diverse cholesterol gene signatures in colon cancer
Source: Front Genet. 2024 Nov 29;15:1410353. doi: 10.3389/fgene.2024.1410353 (PMC11638177; doi:10.3389/fgene.2024.1410353)
Supplement: Supplementary file 1 [file DataSheet1.pdf]

Supplementary Material

1 Supplementary Data

RNA-seq raw counts were retrieved from The Cancer Genome Atlas (TCGA) (https://portal.gdc.cancer.gov/projects), 456 Colon cancer samples and 41 adjacent-normal tissues with survival information, age, gender, and stage were included in analysis. The three Gene Expression Omnibus microarray datasets (https://www.ncbi.nlm.nih.gov/geo/) were used for external validation cohorts: GSE17538 (N=232), GSE33113 (N=90), and GSE39582 (N=566).

2 Supplementary Figures and Tables

2.1 Supplementary Figures

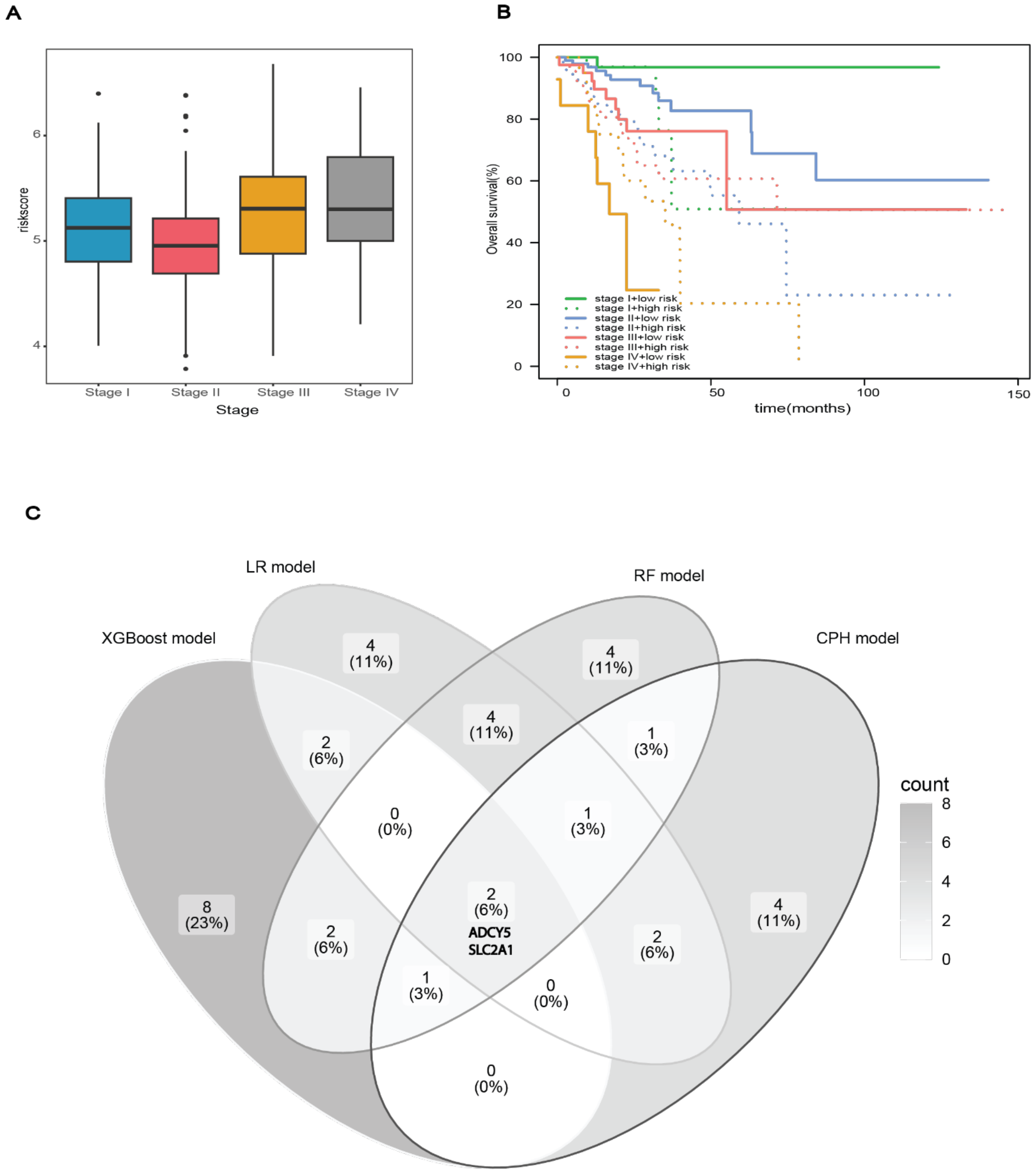

Supplemental Figure 1. Data analysis of TCGA-COAD and overlapped genes in four models. A) The boxplot of stage in TCGA-COAD; B) The overall survival of stage in low and high risk group in TCGA-COAD; C) Overlapped genes in CPH model and ML models.

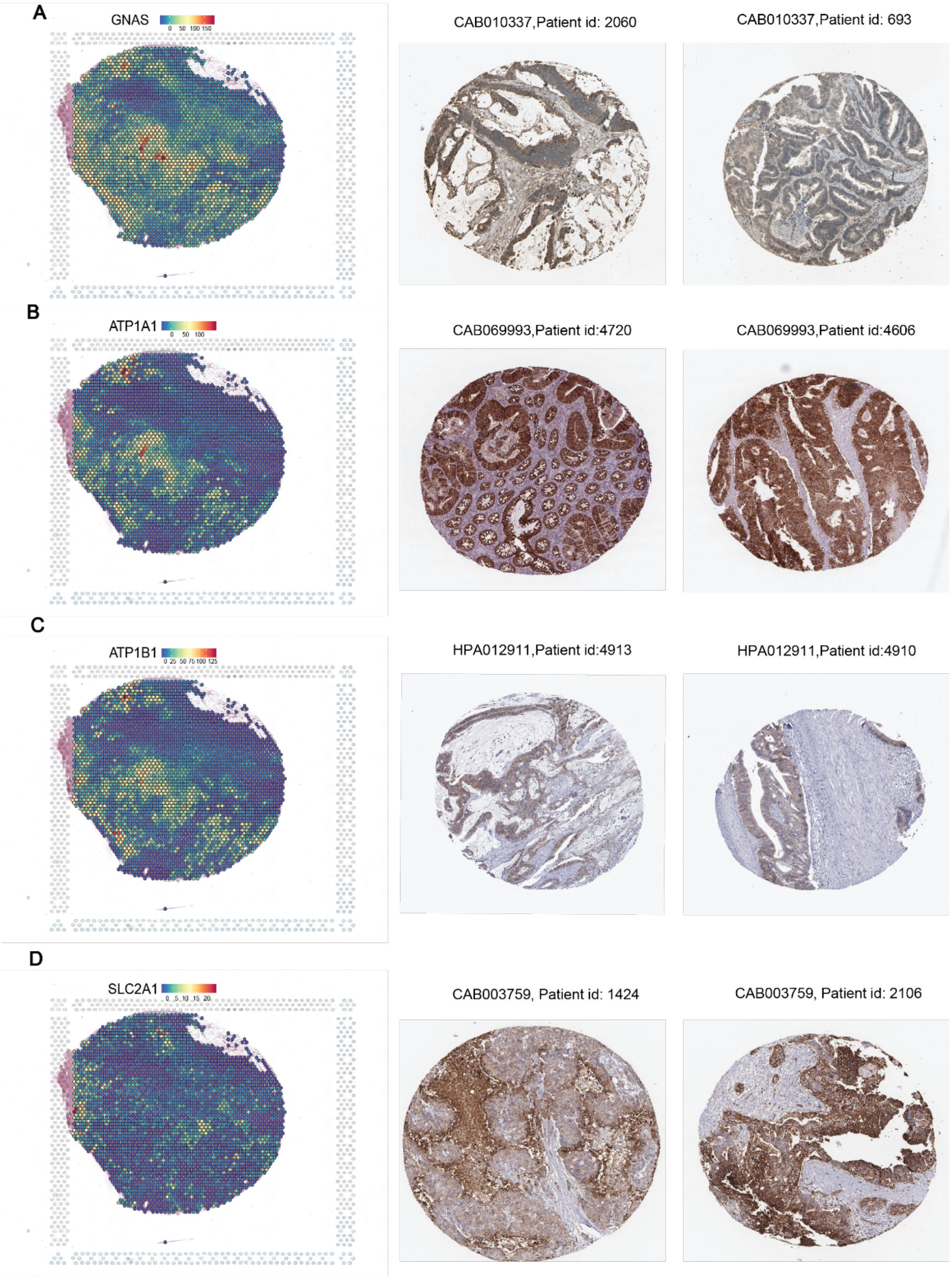

Supplemental Figure 2. Gene spatial visualization and protein expression atlas. A) GNAS gene spatial visualization and two GNAS protein expression atlas with patient id; B) ATP1A1 gene spatial visualization and two ATP1A1 protein expression atlas with patient id; C) ATP1B1 gene spatial visualization and two ATP1B1 protein expression atlas with patient id; D) SLC2A1 gene spatial visualization and two SLC2A1 protein expression atlas with patient id.

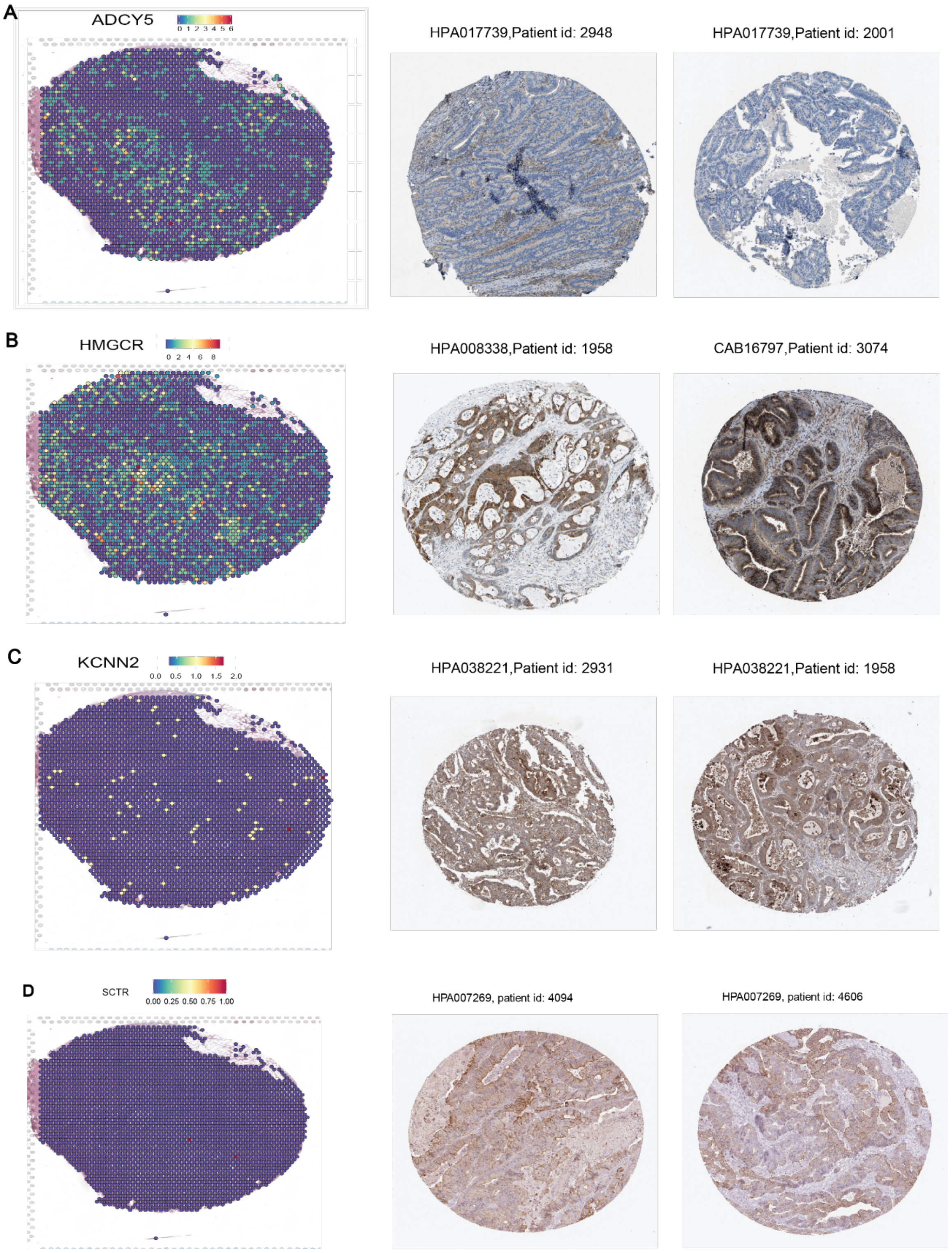

Supplemental Figure 3. Gene spatial visualization and protein expression atlas. A) ADCY5 gene spatial visualization and two ADCY5 protein expression atlas with patient id; B) HMGCR gene spatial visualization and two HMGCR protein expression atlas with patient id; C) KCNN2 gene spatial visualization and two KCNN2 protein expression atlas with patient id; D) SCTR gene spatial visualization and two SCTR protein expression atlas with patient id.

Supplementary Table 1. Adjusted p-value for multiple comparison testing in Univariate CPH model

gene	p-value	BH adjusted p-value	Bonferroni p-value	HR
ADCY5	0.018	0.0436	0.180	1.3245(1.0494, 1.6716)
FXYD2	0.0342	0.0436	0.342	1.6132(1.0362, 2.5113)
CA2	0.0349	0.0436	0.349	0.885(0.7899, 0.9914)
ABCB4	0.0047	0.0436	0.047	1.7529(1.1879, 2.5866)
SLC2A1	0.0393	0.0436	0.393	1.2083(1.0093, 1.4464)
SLC10A2	0.0153	0.0436	0.153	1.8099(1.1207, 2.9228)
UGT2B15	0.0383	0.0436	0.383	0.8512(0.731, 0.9913)
UGT2A3	0.0436	0.0436	0.436	0.8864(0.7884, 0.9965)
SLC51B	0.0317	0.0436	0.317	0.86(0.7495, 0.9869)
ADCY4	0.0137	0.0436	0.137	1.4642(1.0812, 1.9829)

Supplementary Table 2. The list of selected genes in models

XGBoost	LR	RF	CPH	Eight genes
**ADCY5**	**ADCY5**	**ADCY5**	**ADCY5**	SLC10A2
**SLC2A1**	**SLC2A1**	**SLC2A1**	**SLC2A1**	UTS2
UGT2A3	SLC10A2	UGT2A3	UGT2A3	FGF2
SLC27A5	SCTR	SLC10A2	SLC10A2	UCN
SCTR	GNAS	SLC27A5	ABCB4	IL1RL2
GNAS	ABCB4	CYP7A1	SLC51B	ESM1
ABCC2	CYP7A1	SLC22A7	ADCY4	ADIPOQ
ABCC4	SLC22A7	SLCO1B1	CA2	VIP
AQP9	SLCO1B1	SULT2A1	FXYD2	
ATP1A1	SULT2A1	SLC51B	UGT2B15	
ATP1A4	ABCG5	ATP1A1		
ATP1B1	ADCY9	ATP1B3		
BAAT	FXYD2	EPHX1		
HMGCR	NCEH1	SLCO1A2		
KCNN2	UGT1A6	SLCO1B3		

Supplementary Table 3. Patient characteristics in XGBoost Model (TCGA-COAD)

Characteristic	Overall	high-risk group	low-risk group	p-value[2]
	N =456[1]	N =228[1]	N =228[1]	
**Stage**				**<0.001*****
Stage I	75(17%)	38(17%)	37(17%)	
Stage II	176(40%)	63(28%)	113(51%)	
Stage III	128(29%)	79(36%)	49(22%)	
Stage IV	64(14%)	42(19%)	22(10.0%)	
**Age**	66.92(13.09)	65.85(13.66)	67.99(12.43)	0.138
**Gender**				0.188
Female	214(47%)	114(50%)	100(44%)	
Male	240(53%)	113(50%)	127(56%)	

Note: [1] n (%); Mean (SD); [2] Pearson's Chi-squared test; Wilcoxon rank sum test.

Supplementary Table 4. Comparison of model performance metrics for 3-year RFS

Test datasets	XGBoost	Random Forest	LASSO	Cox proportional hazards
**GSE33113**	AUC= 0.664<br>PRAUC= 0.268<br>Sensitivity= 0.765<br>Specificity= 0.569<br>F1= 0.426	AUC= 0.492<br>PRAUC= 0.17<br>Sensitivity= 0.529<br>Specificity= 0.514<br>F1= 0.295	AUC= 0.596<br>PRAUC= 0.239<br>Sensitivity= 0.647<br>Specificity= 0.542<br>F1= 0.361	AUC= 0.598<br>PRAUC= 0.225<br>Sensitivity= 0.588<br>Specificity= 0.528<br>F1= 0.328
**GSE39582**	AUC= 0.565<br>PRAUC= 0.338<br>Sensitivity= 0.558<br>Specificity= 0.524<br>F1= 0.401	AUC= 0.574<br>PRAUC= 0.369<br>Sensitivity= 0.571<br>Specificity= 0.529<br>F1= 0.41	AUC= 0.528<br>PRAUC= 0.302<br>Sensitivity= 0.519<br>Specificity= 0.509<br>F1= 0.373	AUC= 0.581<br>PRAUC= 0.356<br>Sensitivity= 0.564<br>Specificity= 0.526<br>F1= 0.406
**GSE17538**	AUC= 0.599<br>PRAUC= 0.341<br>Sensitivity= 0.604<br>Specificity= 0.533<br>F1= 0.392	AUC= 0.509<br>PRAUC= 0.25<br>Sensitivity= 0.542<br>Specificity= 0.513<br>F1= 0.351	AUC= 0.485<br>PRAUC= 0.264<br>Sensitivity= 0.479<br>Specificity= 0.493<br>F1= 0.311	AUC= 0.584<br>PRAUC= 0.286<br>Sensitivity= 0.625<br>Specificity= 0.539<br>F1= 0.405

Supplementary Table 5. Performance metrics of the XGBoost model on the validation datasets after including clinical covariates.

	**3 year**	**5 year**
**GSE33113 (XGBoost)**	AUC=0.592<br>PRAUC= 0.490<br>Sens= 0.580<br>Spec= 0.590<br>F1= 0.611	AUC= 0.717<br>PRAUC= 0.894<br>Sens= 0.790<br>Spec= 0.571<br>F1= 0.469
**GSE39582 (XGBoost)**	AUC= 0.632<br>PRAUC= 0.464<br>Sens= 0.570<br>Spec= 0.628<br>F1= 0.647	AUC= 0.625<br>PRAUC= 0.586<br>Sens= 0.586<br>Spec= 0.596<br>F1= 0.606
**GSE17538 (XGBoost)**	AUC= 0.734<br>PRAUC= 0.629<br>Sens= 0.598<br>Spec= 0.708<br>F1= 0.692	AUC= 0.747<br>PRAUC= 0.777<br>Sens=0.646<br>Spec= 0.635<br>F1= 0.633
**GSE33113 (Age)**	AUC=0.608<br>PRAUC= 0.514<br>Sens= 0.560<br>Spec= 0.564<br>F1= 0.590	AUC= 0.638<br>PRAUC= 0.862<br>Sens= 0.684<br>Spec= 0.543<br>F1= 0.406
**GSE39582 (Age)**	AUC= 0.503<br>PRAUC= 0.365<br>Sens= 0.513<br>Spec= 0.481<br>F1= 0.576	AUC= 0.518<br>PRAUC= 0.504<br>Sens= 0.530<br>Spec= 0.468<br>F1= 0.533
**GSE17538 (Age)**	AUC= 0.432<br>PRAUC= 0.304<br>Sens= 0.477<br>Spec= 0.354<br>F1= 0.540	AUC= 0.448<br>PRAUC= 0.500<br>Sens= 0.479<br>Spec= 0.462<br>F1= 0.465
**GSE33113 (Stage)**	NA	NA
**GSE39582 (Stage)**	AUC= 0.713<br>PRAUC= 0.442<br>Sens= 0.636<br>Spec= 0.699<br>F1= 0.710	AUC= 0.697<br>PRAUC= 0.528<br>Sens= 0.636<br>Spec= 0.667<br>F1= 0.661
**GSE17538 (Stage)**	AUC= 0.759<br>PRAUC= 0.313<br>Sens= 0.542<br>Spec= 0.792<br>F1= 0.663	AUC= 0.764<br>PRAUC= 0.444<br>Sens= 0.583<br>Spec= 0.788<br>F1= 0.644
